# The genus *Eunotia* Ehrenb. (Bacillariophyta) in the Cheremsky Nature Reserve, Ukrainian Polissya, and refined terminology relevant to the raphe system morphology

**DOI:** 10.3897/phytokeys.128.35566

**Published:** 2019-07-23

**Authors:** Lyudmila N. Bukhtiyarova

**Affiliations:** 1 Institute for Evolutionary Ecology, National Academy of Sciences of Ukraine, Acad. Lebedev str. 37, 03143 Kyiv, Ukraine Institute for Evolutionary Ecology, National Academy of Sciences of Ukraine Kyiv Ukraine

**Keywords:** *
Eunotia
*, functional morphology, mirror-symmetric, mantle-offset, brevisslit raphe system, taxonomy, rare species, distribution

## Abstract

Numerous species of *Eunotia* Ehrenb., widely distributed in the world flora, prefer acidic, dystrophic or oligotrophic freshwater habitats with low conductivity and usually occur in epiphytic or epilithic hydrotopes. In Ukraine, only 32 species and eight varieties of *Eunotia* were known until this study. For the first time, 9 more species have been recorded mainly from the Cheremsky Nature Reserve, located in Ukrainian Polissya. New findings include 2 species widely distributed in the world flora on most continents and 7 rare species known from several locations, among them *E.genuflexa*, *E.jarensis* and *E.ruzickae*, which are probably European endemics as they have not been reported from other continents. For the present time in the Cheremsky Nature Reserve, the 20 species recorded here, the highest species richness of *Eunotia* in Ukraine, bring the total number of *Eunotia* in Ukraine to 41 species, which comprises only 7% of *Eunotia* species in the world flora. This is indirect evidence of insufficient investigation of the wetlands in Ukraine where *Eunotia* usually is represented with high species richness. Several definitions are suggested to describe morphological features that are peculiar to the diatom frustule particular to the *Eunotia* species. The genus Eunotia possesses a mirror-symmetric, *mantle-offset*, *brevisslit raphe system*, which may or may not have *terminal raphe fissures*. Morphological analysis provided in this study revealed the absence of terminal raphe fissures for many species of *Eunotia*. Instead, the distal ends of the raphe slits finish on the outer valve surface by funnel holes, sometimes pore-like ones, connected with the helictoglossae. However, in the literature those distal ends of the raphe slits were described erroneously as terminal raphe fissures. For the first time different types of raphe system are grounded. Two species *Eunotiaimplicata* Nörpel-Schempp et al. in Alles et al. and *Eunotiaincisa* W. Smith ex Gregory were lectotypified.

## Introduction

**Figures 1, 2.**
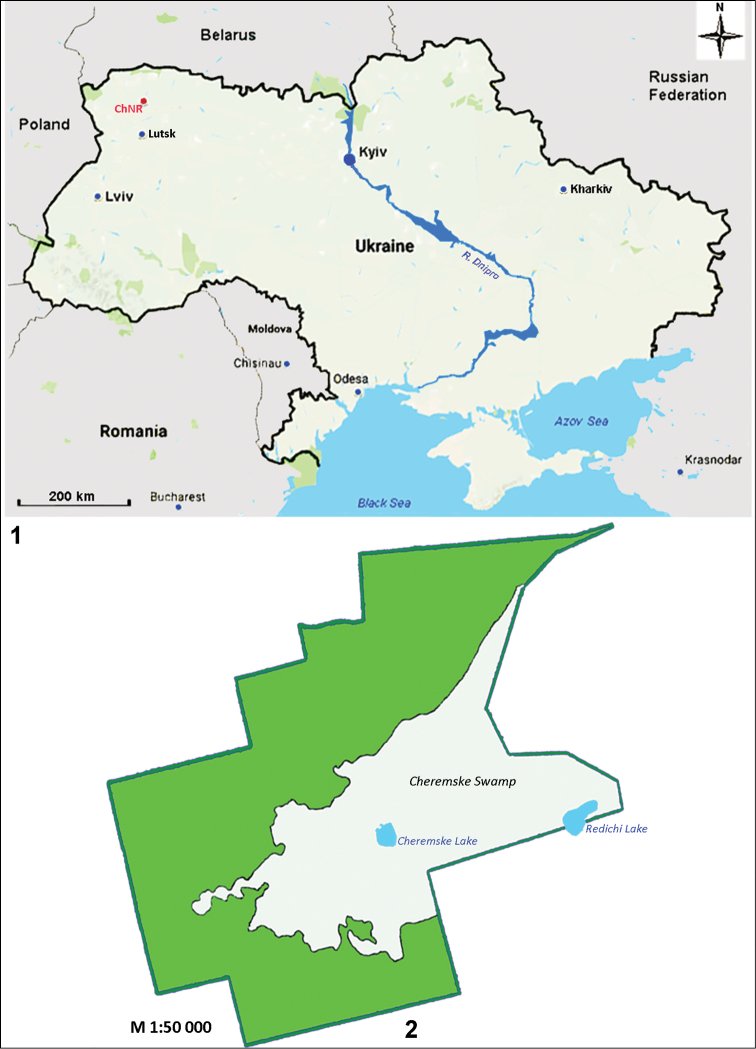
Location of the studied area **1** The Cheremsky Nature Reserve on the map of Ukraine, indicated by red dot **2** map-scheme of the Cheremsky Nature Reserve.

The Cheremsky Nature Reserve, located in Ukrainian Polissya, Volyn region, in the interfluve of Stokhid and Veselukha rivers, occupies about 3 thousand ha. The reserve includes large areas of untouched forests and unique wetlands which take up about 34% of the territory (Figs 1, 2). An eumesotrophic Cheremske bog (total area of about one thousand ha, peat deposition up to 10 m), relates to peripheral-oligotrophic type of development characteristic of bogs formed in lake-like basins. Two lakes within the Cheremsky Nature Reserve Cheremske (7,7 ha, max. depth 7 m) and Redychi (14,0 ha, max. depth 4,5 m) originate from glacial-karst (Konischuk and Didukh 2004, Konishchuk 2005).

Previous studies of the Bacillariophyta from the Cheremsky Nature Reserve revealed high species richness with 84 species reported by Bukhtiyarova (2008) and 57 taxa by Malakhov et al. (2017). The mesotrophic bogs contain a moderate quantity of nutrients that facilitate the development of a specific and rich diatom flora. The most species-rich genera in such habitats typically include *Eunotia* Ehrenb. and *Pinnularia* Ehrenb. Indeed, many of the diatom taxa recorded for the first time in Ukraine were from the Cheremsky Nature Reserve and included: *Eunotiasilvahercynia* Nörpel et al. in Alles et al. (1991), *Pinnulariacomplexa*Krammer (2000), *P.lokana*Krammer (2000), P.nobilisvar.regularisKrammer (2000), *P.parvulissima*Krammer (2000), *P.polyonka* (Bréb.) W. Smith (1856), *P.rhombarea* Krammer in Metzeltin and Lange-Bertalot (1998), P.stomatophoravar.irregularisKrammer (2000), *P.stidolphii*Krammer (2000), *P.subanglica*Krammer (2000), *P.subrupestris*Krammer (1992) and its variety P.subrupestrisvar.cruciataKrammer (2000) (Bukhtiyarova 2008, 2009b); *Eunotiamyrmica* Lange-Bert. in Lange-Bertalot et al. (2011) (Malakhov et al. 2017). In the Cheremsky Nature Reserve only 9 species of *Eunotia* were known from the Cheremske and Redychi lakes prior to this study.

As a part of documenting the *Eunotia* taxa it was necessary to describe the raphe system’s particular properties which have taxonomical value on species rank of taxonomy. In recently published terminological glossary the following definition for the raphe was proposed: “Raphe (Lat.) – an elongated slit or pair of slits through the valve wall. When a pair of slits is present each individual slit is a branch of the raphe” (Gogorev et al. 2018: p. 299), which does not include the position of the raphe system in the diatom frustule hierarchic structure and any of its functions. In another glossary about the same definition appears: “The raphe system is composed of one or two slits, or fissures, that penetrate the valve of some diatoms. If two slits are present, each is called a branch of the raphe. Branches may be separated by a silica thickening called the central nodule” and it was indicated that raphe allows diatom cells to move (Diatoms of North America 2019). In the latest one the possible raphe position on the valve is defined as axial (along the apical axis), eccentric (along one margin) or circumferential (around the whole margin of the valve) none of which does not specify raphe position on the valves of the *Eunotia* species. In both definitions several structural elements of the raphe system are omitted. Moreover, in the second, no distinction is made between the raphe slits and fissures, as both terms look like synonyms but they define different elements of the raphe system. The *Eunotia* taxa possess unique raphe system the morphology of which has not been studied yet in detail.

This study provides detailed information on the species of *Eunotia* Ehrenb. found in the Cheremsky Nature Reserve, including rare species recorded in Ukraine for the first time. Revised terminology to highlight morphological features of the raphe system relevant to the *Eunotia* species is also suggested.

## Materials and methods

In 2003–2004 O. Petlyovany collected epiphytic samples of algae from mosses in the Volyn region, Manevichsky district, the Cheremsky Nature Reserve mainly from the lakes Cheremske and Redychi, both from open waters and marshy locations. Sample numbers correspond to those from the Algoteca of M.G. Kholodny Institute of Botany, National Academy of Sciences of Ukraine – the largest phycological collection in Ukraine.

**30586** The Cheremsky Nature Reserve, wetland area, Lake Cheremske, epiphyton on *Sphagnum* sp. 06/18/2003.

**30588** The Cheremsky Nature Reserve, tract Obkopane, ditch, epiphyton on *Sphagnum* sp. 06/18/2003.

**30599** The Cheremsky Nature Reserve, tract Obkopane, Lake Redychi, epiphyton on *Sphagnum* sp. 06/19/2003.

**30635** The Cheremsky Nature Reserve, tract Obkopane, Lake Redychi, epiphyton on *Fontinalis* sp. 08/14/2004.

**30637** The Cheremsky Nature Reserve, tract Obkopane, Lake Redychi, epiphyton on *Sphagnum* sp. 08/14/2004.

**30640** The Cheremsky Nature Reserve, tract Obkopane, Lake Redychi, wetland area, epiphyton on *Sphagnum* sp. 08/14/2004.

In accordance with Malakhov et al. (2017) hydrochemical parameters of these lakes are very similar. Lake Cheremske: conductivity – 70 µS/cm, pH – 6.16–6.6, dissolved O_2_ – 9.7 mg/L, NH_4_^+^ – 0.22 mg/L, NO_2_- < 0.01 mg/L, NO_3_- < 0.01 mg/L, PO_4_^3–^< DL mg/L, Fe ^3+^ – 1.2 mg/L.

Lake Redychi: conductivity – 63 µS/cm, pH – 6.46, dissolved O_2_ – 9.5 mg/L, NH_4_^+^ – 0.20 mg/L, NO_2_- < 0.01 mg/L, NO_3_- < 0.01 mg/L, PO_4_^3–^< DL^*^ mg/L, Fe ^3+^ – 1.1 mg/L.

Two samples from other locations in Ukrainian Polissya collected by O.V. Kovalenko have been also studied.

**27835** Zhytomyr region, Chervonoarmeisky district, swamp, dark films among mosses. 07/15/1983.

**27895** Volyn region, Vladimir-Volyn district, near village Fedorovka, Western Bug River, floodplain basin, benthos. 07/21/1983.

Organic matter was removed by cold burning with concentrated sulfuric acid and cleaned materials were rinsed several times with distilled water (Wasser et al. 1989). Permanent slides with cleaned materials were mounted in Naphrax (R.1 = 1.7). Diatom species were examined with an Olympus BX 51 light microscope (LM) using a 100× oil immersion PlanAchromat objective. The fine structure of the diatom frustules was examined with a scanning electron microscope JEOL 6060LA. The micrographs were obtained with Canon EOS 600 D digital camera using program Helicon Remote (version 3.6.2 w).

Size ranges were based on measurements typically several, sometimes single valves as all species were found in very limited numbers. Therefore the size ranges from the relevant literature were included in the species description.

In many diatom species with bipolar symmetry including *Eunotia* different morphometric data present at different valve parts, e.g. width, striae density in 10 µm etc., their dimension can be helpful in the species correct identification.

**Figure 3.**
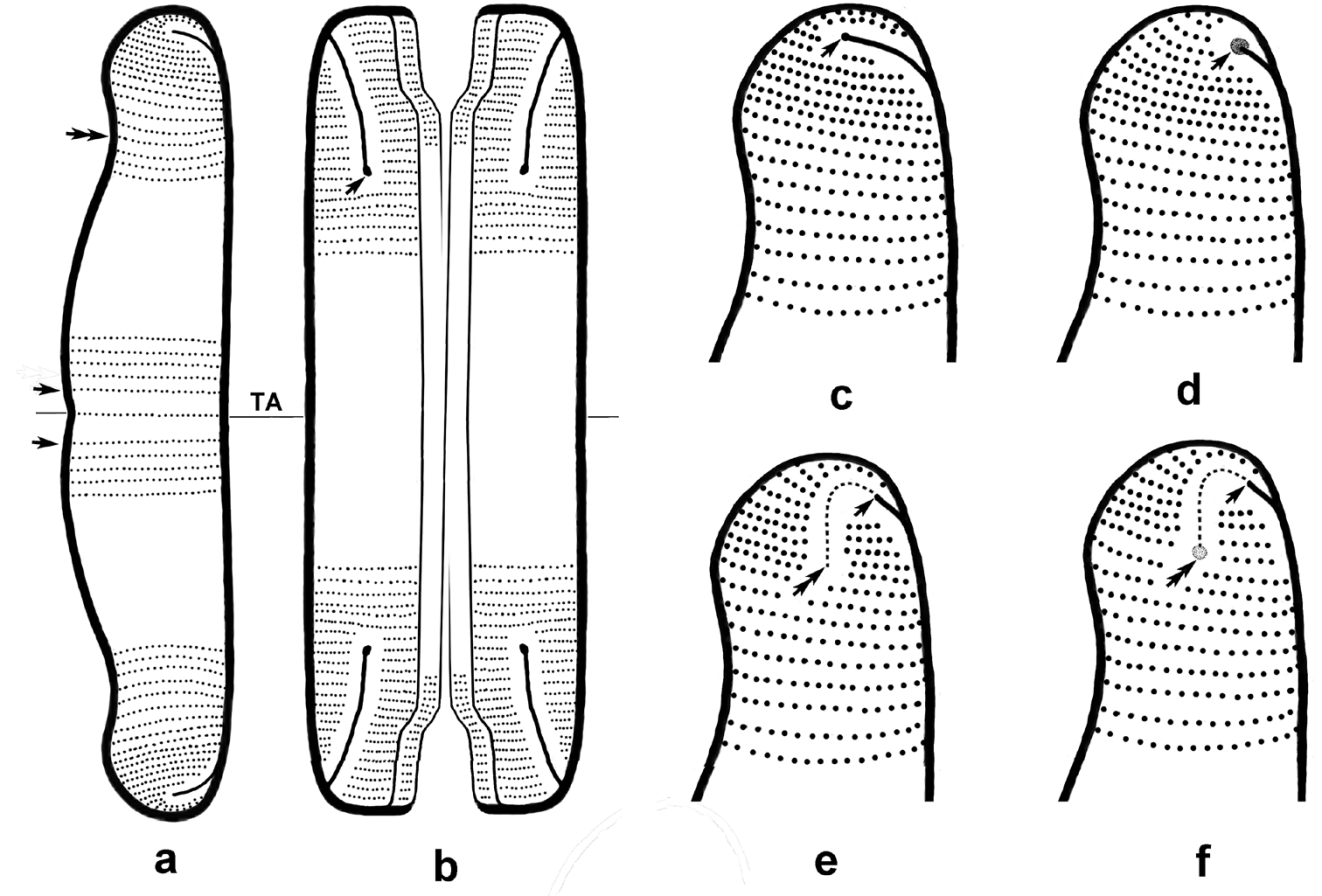
Scheme of the mirror-symmetric, mantle-offset, brevisslit raphe system in frustule of the genus *Eunotia* Ehrenb. **A** valve view, different parts of valve. TA – transapical axis. Central part – between the arrows, middle part – between upper and double arrows, the beginning of valve pole – double arrow **B** girdle view, arrow – central raphe pore **C, D** raphe system without terminal fissures **C** arrow – a pore outer at distal end of slit, that finishes at the middle of valve pole **D** arrow – a funnel-like outer at distal end of slit, that finishes at the venral corner of valve pole **E, F** raphe system with terminal fissures – dotted lines between arrows, arrow – distal end of a slit **E** double arrow – the end of terminal fissure **F** double arrow – lacuna at the end of terminal fissure. (Original by L. Bukhtiyarova)

Central valve part – valve part on both sides from the transapical axis where the measuring parameter has different value comparing with other valve parts (Fig. 3a, between the arrows).

Middle valve part – valve part between central part and the beginning of valve pole (Fig. 3a, between the upper and double arrows).

Valve pole – distal valve part from its narrowest part or from the beginning of a valve narrowing to the distal end (Fig. 3a, from the double arrow to distal end of a valve).

For the algae flora of Ukraine a 'very rare species' is defined as one recorded in 1–5 localities and 'rare species' – in < 10 localities within the country territory (Palamar’-Mordvintseva et al. 1998). A 'rare species in the world flora' is defined here as one recorded in fewer than 10 localities and restricted to 1–2 continents.

**Abbreviations.** Morphometric data example: length 45–97 µm, width c6–7, m9–12, p3–5 µm; striae density c12–16, p18–20 in 10 µm.

**c5–7** the data for the central valve part.

**m9–12** the data for the middle valve part.

**p18–20** the data for the valve pole.

* species recorded for the first time in Ukraine.

^ rare species in the world flora.

## Results

### Morphology and refined terminology

The revised definitions suggested here for some widely used terms and new ones are grounded on the concept of functional morphology of the diatom frustule (Bukhtiyarova 2009a, 2019) which includes a number of theses, in particular the division of all diatom frustule structures on the basic elements and functional units. Thus, this concept allows us to describe any morphological structure on a common universal basis.

Because physical-chemical properties of any material depend on the size of the particles it is compounded from, it was suggested to introduce a size scale in all the definitions of the diatom frustule structures (Bukhtiyarova 2009a).

**The basic element of the diatom frustule (db-element)** is a morphologically detached, homogeneous part of the frustule that possesses special physical-chemical features and provides primary basis for the frustule hierarchical construction. They belong to db-elemets of the diatom frustule as different apertures and cavities in its thickness, regularly repeated and unique silica microelemets (Bukhtiyarova 2009a: figs 1–5).

**Morph of the diatom frustule (df-morph)** is compound structural unit of the diatom frustule that is constructed of db-elements and/or structural units of lower orders, realizes particular functions in the diatom organism and has its own evolution (Bukhtiyarova 2009a).

The refined definitions capture raphe system as a functional unit of the diatom frustule, its different db-elements and peculiar properties relevant to *Eunotia* species. For the first time different raphe system types are grounded.

**Raphe system** – a unique compound micro df-morph of second (first) order in the diatom frustule with bipolar symmetry that consists of one or two slits, which penetrate the valve thickness, and may include additional df-morphs (central nodule, helictoglossae, tube) and/or db-elements (terminal fissures, central pores and others). One of the functions of the raphe system is active moving of the diatom unicellular organism. For the species that have an attached mode of life other functions can be performed, e.g. an attachment to substrate or between neighboring cells in a colony.

**Raphe slit** – a unique micro db-element in the shape of uniformly narrowed through opening of different profile into the valve thickness and noticeable length relative to the valve length.

**Central raphe pore** – a unique micro db-element, through hole with usually a different shape on the inner and outer valve surfaces at the proximal end of the raphe slit (Fig. 3b, arrow).

**Terminal raphe fissure (tr-fissure)** – a unique micro db-element in the shape of uniformly narrowed non-through notch continuing distal end of the raphe slit on external valve surface only (Figs 3e, f).

**Figures 4–6b.**
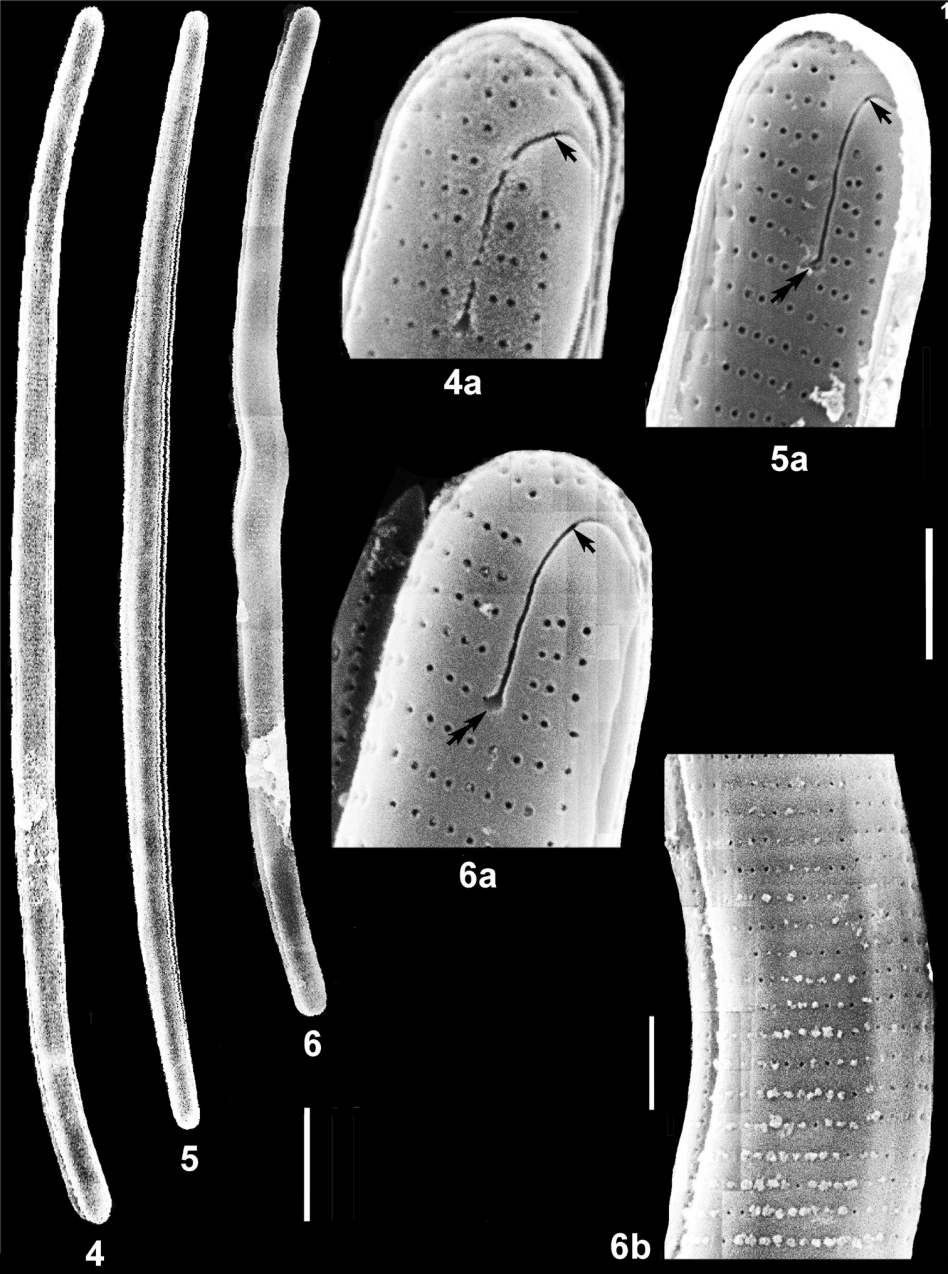
New in Ukraine species *Eunotiagenuflexa* Nörpel-Schempp in Lange-Bertalot and Metzeltin, outside valve surface, SEM. Arrows indicate distal ends of raphe slits, double arrows – funnel-like lacunae, terminal fissures are between the arrows. Scale bars: 10 µm (**4–6**); 2 µm (**4a, 5a, 6a**); 1 µm (**6b**).

**Terminal raphe fissure with lacuna** – kind of fissure that finishes on distal end by lacuna (Figs 3f, 4a, 5a, 6a, 8a).

**Figures 7–12a.**
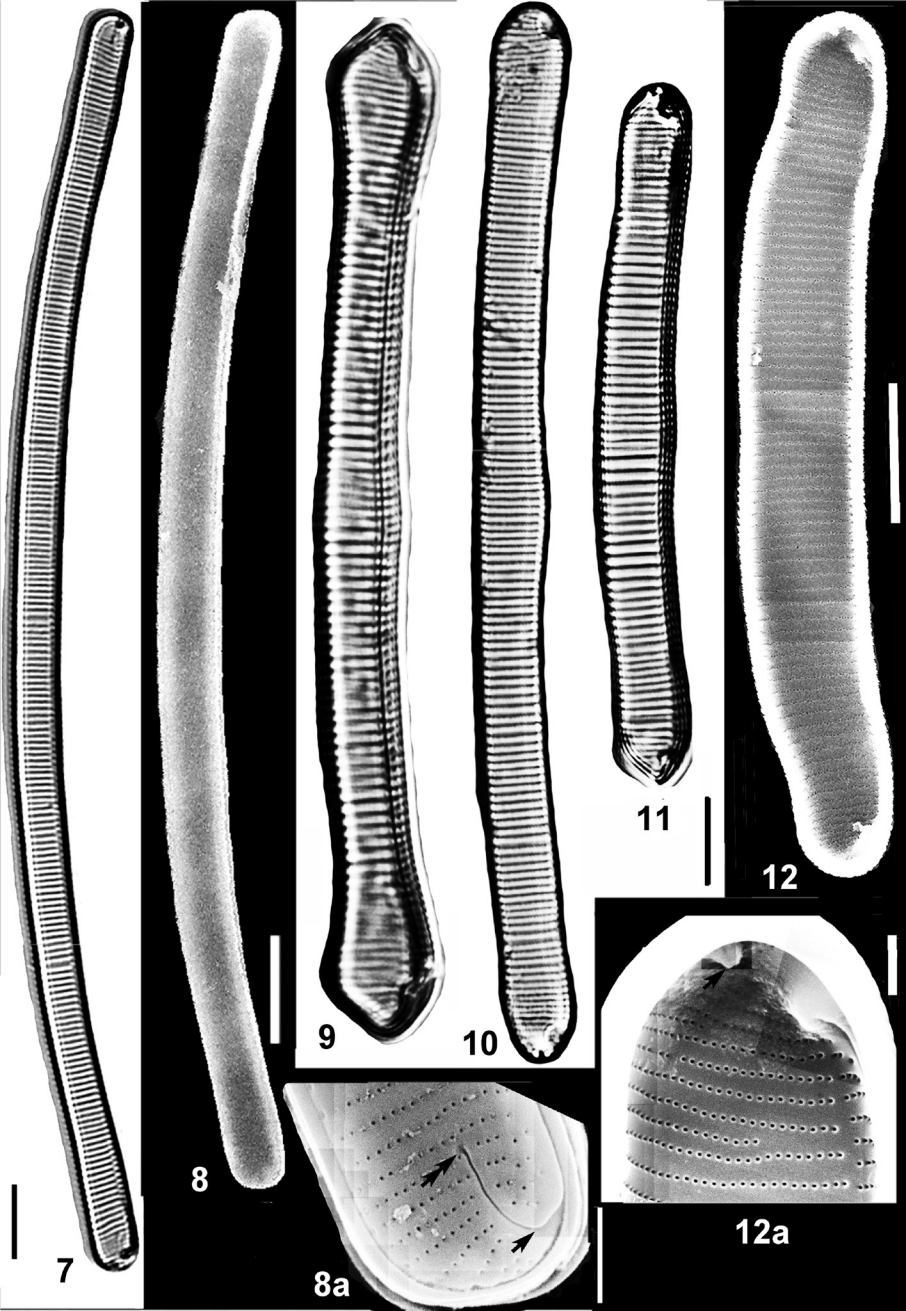
New and rare in Ukraine species of *Eunotia* Ehrenb. from the Cheremsky Nature Reserve. **7–8a***Eunotiajulma* Lange-Bert. in Lange-Bertalot et al., arrow indicates distal end of raphe slit, double arrow – funnel-like lacuna, terminal fissure is between the arrows **9***Eunotia* sp. 1 (cf *E.formica* Ehrenb.) **10, 11***Eunotiaformicina* Lange-Bert. in Lange-Bertalot et al. **12, 12a***Eunotiajarensis* Lange-Bert. et al., arrow indicates rimoportula. Figs **7, 9–11** LM **8, 8a** outside valve surface **12, 12a** inside valve surface, SEM. Scale bars: 10 µm (**7–17**); 3 µm (**8a**); 2 µm (**12a**).

**Lacuna** – a unique micro or nano- db-element, non-through hollow of different shape and location on outer or inner valve surface.

In the genus *Eunotia* the lacunae of the raphe terminal fissures (rtf-lacuna) usually have a dish or funnel-like shape of about 100 nm in diameter (Figs 3f, 4a, 5a, 6a, 8a, double arrows).

**Figures 13–27.**
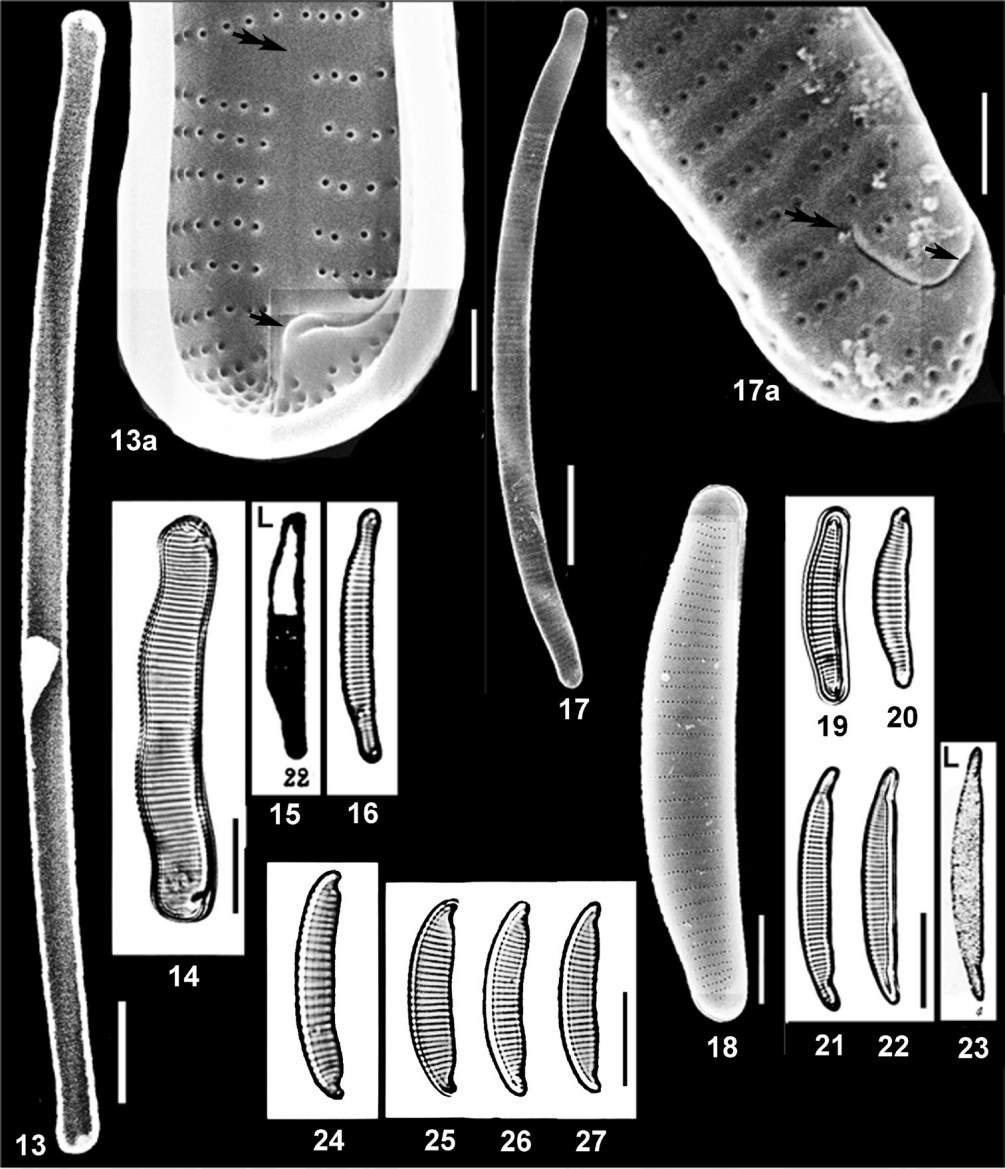
New and rare in Ukraine species of *Eunotia* Ehrenb. from Ukrainian Polissya and the Lectotypes of two *Eunotia* species. **13, 13a***Eunotiapseudoflexuosa* Hust., arrow indicates rimoportula at the middle of pole where raphe slit distal end finishes on the outer valve surface, double arrow – position of terminal fissure distal end on the outer valve surface, between the arrows – hyaline field from inside valve surface where the terminal fissure is located on the outer valve surface **14***Eunotiaruzickae* Bílý & Marvan **15, 16***Eunotiaimplicata* Nörpel-Schempp et al. **Lectotype**: 15 = Eunotiaimpressavar.angusta Grunow in Van Heurck 1881: pl. 33/fig. 22, designated here **17, 17a***Eunotia* sp. 2 (cf.mongolica Kulikovskiy et al.) **18–20***Eunotia* sp. 3 (cf. *Eunotiapaludosa* Grunow) **21–23***Eunotiaincisa* W. Smith ex Gregory. **Lectotype**: 23 = *Eunotiaincisa* W. Smith ex Gregory 1854: pl. 4/fig. 4, designated here **24***Eunotia* sp. 4 (cf. *Eunotiaintermedia* (Krasske ex Hustedt) Nörpel & Lange-Bert.) **25–27***Eunotia* sp. 5 (cf. *Eunotiameridiana* Metzeltin & Lange-Bert.). Figs **13, 17, 18** SEM: **13, 13a** inside valve surface **17, 17a, 18** outside valve surface; **19–28** LM. Scale bars: 10 µm (**13–17, 19–27**); 5 µm (**18**); 1 µm (**13a, 17a**).

**Helictoglossa** – a unique siliceous hyaline micro df-morph of first order on the internal valve surface usually in the shape of truncated cylinder or compressed (relative to the raphe slit) asymmetric frustum with smoothly roused up side bearing fissure at the distal end of the raphe slit and abruptly roused opposite side (Figs 13a, 29, 30a, arrows). Any functions of helictoglossae are unstudied, yet their morphology and position allow to suggest that they work like a stopper and may regulate a mucilage mass length of uniform shape that enters into the raphe slit from inside and goes out from the diatom frustule outside.

**Helictoglossa fissure (h-fissure)** – a unique micro db-element in the shape of a uniformly narrowed short non-through notch on helictoglossa continuing distal end of the raphe slit on internal valve surface only.

**Symmetric raphe system** – type of raphe system with straight equal length of the slits and similar accompanied db-elements that are located symmetrically relatively both to the longitudinal and transapical axes of bipolar frustule. Examples of this type of raphe system can be found among species of *Cavinula* D.G. Mann & Stickle in Round et al. (1990), *Hippodonta* Lange-Bertalot et al. (1996), *Navicula* Bory (1822), *Psammothidium* Bukht. & Round (1996) and other diatom genera.

**Mirror-symmetric raphe system** – type of raphe system with equal length and same shaped raphe slits, similar accompanied db-elements that all together are located mirror symmetrically relative to the transapical axis or/and in girdle view of the diatom frustule. Examples of this type of raphe system can be found in species of *Amphora* Ehrenb. ex Kütz. (1844), *Cymbella*Agardh (1830), *Eunotia* and other genera.

**Mantle-offset raphe system** (Lat.) – type of raphe system which partially or completely disposes on the valve mantle. This type of raphe system characterizes the genus *Eunotia*.

**Brevisslit raphe system** (Lat.) – type of raphe system with the slits which disposes only along part of valve length and absent on the rest of it. The examples of this raphe system type can be found in the genera *Actinella* F.W. Lewis (1864), *Eunotia, Rhoicospenia*Grunow (1860) and in others.

Thus, species of the genus *Eunotia* possess of mirror-symmetric, mantle-offset, brevisslit raphe system.

**Basal striae** – type of striae in which the areolae and all additional db-elements accompanying them occupy interstria height in whole (Bukhtiyarova 2015: figs 2, 10–15), or by other words, the valve thickness completely.

**Distant striae** – kind of striae which occupy two or more times less area than interstria area (Bukhtiyarova 2015: figs 4, 6, 8, 9–13, 17).

The terms **proportional**, **packed**, **distant kinds of striae** have been defined on the ratio between stria and interstria areas (Bukhtiyarova 2015).

### Taxonomy

In the hydrotopes of the Cheremsky Nature Reserve the following species of the genus *Eunotia* were recorded.

#### Eunotia dorofeyukiae

Taxon classificationPlantaeEunotialesEunotiaceae

Lange-Bert. & Kulikovskiy in Kulikovskiy et al. 2010b: p. 29, 65, pl. 20/figs 1–6. *^

913ced8f-3b2f-5967-aca7-fadf1932faac

**Figures 28–35.**
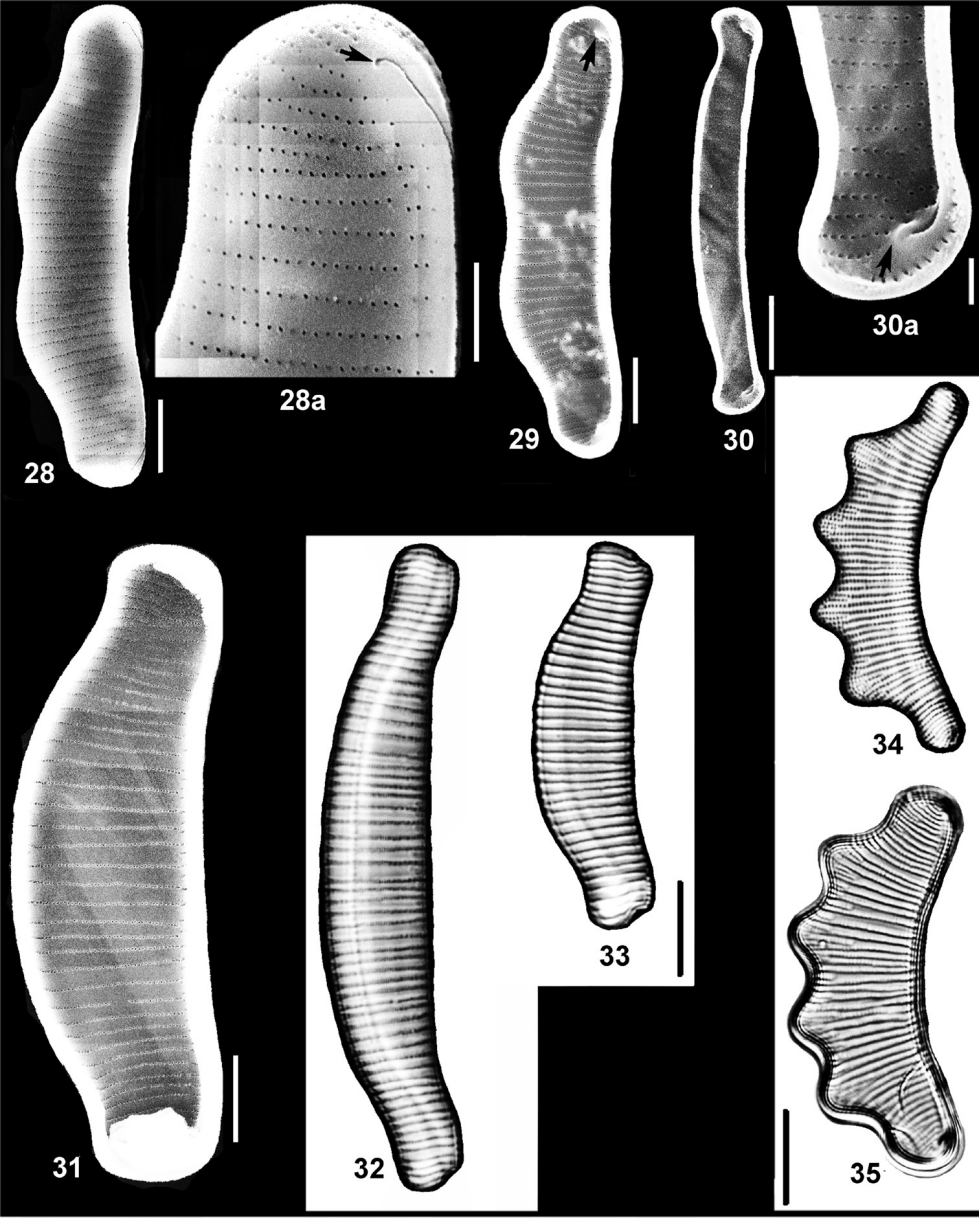
Species of *Eunotia* Ehrenb. from the Cheremsky Nature Reserve. **28, 28a, 29***Eunotiadorofeyukiae* Lange-Bert. & Kulikovskiy **28a** arrow indicates pore outer on the raphe slit distal end **29** arrow indicates rimoportula at the venral corner of pole where raphe slit distal end finishes on the outer valve surface **30, 30a***Eunotianeocompacta* S. Mayama **30a** arrow indicates rimoportula at the venral corner of pole, absence of a hyaline field indicates absence of terminal fissure **31***Eunotiapraerupta* Ehrenb. **32***Eunotia* sp. 6. **33***Eunotia* sp. 7. **34, 35***Eunotiatetraodon* Ehrenb. Figs **28–31** SEM: **28, 28a** outside valve surface; **29–31** inside valve surface; **32–35** LM. Scale bars: 5 µm (**28, 29, 30**); 2 µm (**28a**); 1 µm (**30a**); 10 µm (**31–34**).

Figs 28 , 28a , 29 (SEM)

##### Illustrations.

Krammer and Lange-Bertalot 1991: pl. 143/figs 22–23 (as *Eunotiacircumborealis* Nörpel & Lange-Bert.); Rivera-Rondón and Catalan 2017: pl. 25/figs 7–9.

##### Diagnosis.

Morphometric data: length 33–35 µm, width cm 7, p5–8 µm; striae density c12–14, p18–22 in 10 µm. Lange-Bertalot et al. 2010: length 37–58 µm, width 7.3–8.7 µm, striae density 10–13 in 10 µm.

Frustule bi-symmetric, bipolar, biraphid with mirror-symmetric, mantle-offset, brevisslit type of raphe. Valves dorsiventral, with undulate dorsal margin and weak depression in its central part, slightly concave ventral side and subcapitate broad rounded poles. Striae basal, uniserial, distant, denser at the poles. Areolae small with round outer foramina (Fig. 28a). Raphe system consists of two short filiform slits on ventral valve mantle, distal ends of slits finish on external valve surface on ventral pole corners by small round pore outers (Fig. 28a) connected with small helictoglossae (Fig. 29); tr-fissures absent.

##### Ecology.

Freshwater epiphytic species, often collected on different *Sphagnum* species, occurs in acidic (pH 5.5–5.6), oligotrophic waters with low electric conductivity and buffered by humic acids. The specimens from Type population were collected at 11–13 °C (Kulikovskiy et al. 2010b).

##### Distribution.

ASIA: **Type location**: northern Mongolia, Nur bog (Kulikovskiy et al. 2010b); Russia (Kulikovskiy et al. 2016). EUROPE: Scandinavia (Krammer and Lange-Bertalot 1991 (as *E.circumborealis*); Germany (Kulikovskiy et al. 2010b); Ukraine (present paper); France, Pyrenees, Lake Mariola (Rivera-Rondón and Catalan 2017). AUSTRALIA: Tasmania (M. Guiry in Guiry and Guiry 2019). **In Ukraine.** The Cheremsky Nature Reserve, tract Obkopane, ditch, epiphyton on *Sphagnum* sp.

##### Comments.

Illustrations of this species in Kulikovskiy et al. (2010b: pl. 20/figs 1–6) are not uniform in valve outline.

This species was described from a mountainous region with a harsh climate and was recorded later in a mountain lake in the Pyrenees. In Ukraine it inhabits in a flatland bog.

#### Eunotia formicina

Taxon classificationPlantaeEunotialesEunotiaceae

Lange-Bert. in Lange-Bertalot et al. 2011: p. 105–107, pl. 222/figs 1–7, pl. 223/figs 1–7. *^

913ced8f-3b2f-5967-aca7-fadf1932faac

Figs 10 , 11 (SEM)

Eunotia formica var. elongta Hustedt, 1909
Eunotia formica f. elongata (Hustedt) Ant. Mayer, 1918
Eunotia formica sensu Germain, 1981

##### Holotype.

Lange-Bertalot et al. 2011: pl. 222/fig. 1.

##### Illustrations.

Bąk et al. 2012: p. 131, pl. 11/7 exemplars; Ector et al. 2012: p. 249–250, 3 exemplars; Costa 2015: p. 50, pl. 100/figs 1–5; Marra et al. 2016: fig. 53.

##### Diagnosis.

Morphometric data: length 83–125 µm; width m8, cp9 µm; striae density m9–11, p12–15 in 10 µm. Lange-Bertalot et al. 2011: length 20–170 µm; width c7–10, m6–8 µm; striae density 8–12, p15–16 in 10 µm.

Frustule bi-symmetric, bipolar, biraphid with mirror-symmetric, mantle-offset, brevisslit type of raphe. Valves weakly dorsiventral, with gentle gibbosity in central valve part on ventral side and subcapitate broad rounded poles. Valve mantle high, of about 0.5 of valve width, perpendicular to the valve surface; valve/mantle junction narrow hyaline (see Lange-Bertalot et al. 2011: pl. 223/fig. 4). Striae basal, uniserial, distant, irregularly spaced along the valve; on the mantle additional short intercalar striae (see Lange-Bertalot et al. 2011: pl. 223/fig. 4). Areolae small with round outer foramina. Raphe system consists of two short filiform slits on ventral valve mantle; central raphe pores round; tr-fissures long, widely round, follow the pole outline and finish on dorsal valve margin (see Lange-Bertalot et al. 2011: pl. 223/figs 2, 3, 5–7).

##### Ecology.

Freshwater benthic species, occurs in moderately acidic, dystrophic or oligosaprobic waters (Lange-Bertalot et al. 2011).

##### Distribution.

EUROPE: France, Germany, Netherlands, Poland (M. Gury in Guiry and Guiry 2019); Ukraine (present paper). S. AMERICA: Brasil (Costa 2015, Costa et al. 2017; Marra et al. 2016). **In Ukraine.** The Cheremsky Nature Reserve, tract Obkopane, ditch, epiphyton on *Sphagnum* sp.; Lake Redychi, wetland area, epiphyton on *Sphagnum* sp.

#### Eunotia genuflexa

Taxon classificationPlantaeEunotialesEunotiaceae

Nörpel-Schempp in Lange-Bertalot and Metzeltin 1996: p. 50, pl. 9/figs 14–17. *^

913ced8f-3b2f-5967-aca7-fadf1932faac

Figs 4–6b (SEM)

Eunotia flexuosa f. beta A. Berg, 1939
Eunotioforma genuflexa (Nörpel-Schemp) Kociolek & Burliga in Burliga et al. 2013

##### Illustrations.

Bąk et al. 2012: p. 132, pl. 17/1, 2, 5 exemplars from left to right; Kulikovskiy et al. 2016: p. 121, pl. 20/figs 10–14.

##### Diagnosis.

Morphometric data: length 70–120 µm; width cm2–3, p1.5–2 µm; striae density c20, p23 in 10 µm. Lange-Bertalot and Metzeltin 1996: length 70–160 µm, width 1.5–2.6 µm, striae density 19–23 in 10 µm.

Frustule bi-symmetric, bipolar, biraphid with mirror-symmetric, mantle-offset, brevisslit type of raphe. Valves slightly dorsiventral, with narrowed rounded poles. Striae basal, uniserial, distant, uniformly spaced along the valve (Figs 4a, 5a, 6a, b). Areolae small with round outer foramina. Raphe system consists of two short filiform slits on ventral valve mantle; tr-fissures long, broadly curved, sited on 0.5 of valve width along 4–5 striae, end up by round lacunae (Figs 4a, 5a, 6a).

##### Ecology.

Freshwater benthic species occurs in moderately acidic, dystrophic or oligosaprobic waters. Type location had extremely low concentration of inorganic nutrient and pH = 6.5 (Lange-Bertalot and Metzeltin 1996).

##### Distribution.

EUROPE: **Type location**: Finland, Lake Julma Olkky (Lange-Bertalot and Metzeltin 1996); Poland (Bąk et al. 2012); Russia (Kulikovskiy et al. 2016); Ukraine (present paper). **In Ukraine.** The Cheremsky Nature Reserve, tract Obkopane, Lake Redychi, epiphyton on *Fontinalis* sp.

##### Comments.

Specimens with straight valve outline and subcapitate poles presented in Costa (2015: p. 55, pl. 14/figs 1–11) differ from type population significantly, therefore the author has presented other species under this name. In Bąk et al. (2012) morphology of only three exemplars corresponds to type population.

Type species of the genus *Eunotioforma* Kociolek & Burliga is *Eunotioformamattogrossiana* Kociolek, Burliga & Salomoni (Burliga et al. 2013) has several characters that differ from the genus *Eunotia*: axial area on valve surface (= sternum), large helictoglossae, different number of rimoportulae (2–8 per valve), small granules along all striae which are not present in *E.genuflexa*. Strongly curved long tr-fissures are the only common character with a new genus and it can be found in some other species of *Eunotia*, for instance, *E.flexuosa* (Bréb.) Kütz. (Pavlov and Levkov 2013: pl. 7/fig. 2), *E.macedonica* Pavlov & Levkov (Pavlov and Levkov 2013: pl. 10/fig. 2). The only one character is not enough for transferring of *E.genuflexa* to the genus *Eunotioforma*.

#### Eunotia implicata

Taxon classificationPlantaeEunotialesEunotiaceae

Nörpel-Schempp et al. in Alles et al. 1991: p. 206, pl. 7/figs 19–32. *

913ced8f-3b2f-5967-aca7-fadf1932faac

Figs 15 , 16

Eunotia impressa var. angusta Grunow in Van Heurck, 1881: pl. 33/fig. 22 [Basionym]

##### Lectotype.

Eunotiaimpressavar.angusta Grunow in Van Heurck 1881: pl. 33/fig. 22 (= Fig. 14 here), designated here.

##### Illustrations.

Krammer and Lange-Bertalot 1991: p. 197, pl. 143/figs 1–7; Ortiz-Lerín and Cambra 2007: p. 424, pl. 3/fig. C, pl. 4/figs B, I (SEM); Furey et al. 2011: p. 50, pl. 28/figs 1–8; Lange-Bertalot et al. 2011: p. 119, pl. 97/figs 1–39, pl. 225/figs 16–19; Bąk et al. 2012: p. 133, pl. 16/1, 5 exemplars from left to right; Levkov and Pavlov 2013: p. 25, pl. 56/figs 1–26, pl. 57/figs 4–7 (SEM), pl. 65/figs 1–4 (SEM); Costa 2015: p. 54, pl. 27/figs 1–7, pl. 28/figs 1–5 (SEM); Ector et al. 2015: p. 251, 30 exemplars; Bahls et al. 2018: pl. 111/fig. 15.

##### Diagnosis.

Morphometric data: length 26 µm, width 3.5 µm, striae density c18, p20 in 10 µm. Alles et al. 1991: length 18–30 µm, width 3–5 µm, striae density 14–20 in 10 µm.

Frustule bi-symmetric, bipolar, biraphid with mirror-symmetric, mantle-offset, brevisslit type of raphe. Valves slightly dorsiventral, linear with weakly convex dorsal margin, concave ventral margin and protracted rounded poles. The mantle’s height is equal to about 0.5 of valve width, abruptly perpendicular to the valve surface (see Costa 2015: pl. 28/figs 2, 4). Striae basal, uniserial, distant, uniformly spaced along the valve and compacted at the poles, uninterrupted on dorsal mantle/valve junction and interrupted by sternum on ventral mantle; on dorsal mantle short intercalar striae present (see Costa 2015: pl. 28/figs 2, 4). Areolae small with round outer foramina. Raphe system consists of two short filiform slits on ventral hyaline part of mantle, distal ends of the slits turned to the valve centre under right angle and finish on external valve surface in ventral corner of the poles by small round pores (see Costa 2015: pl. 28/figs 1, 2) connected with helictoglossae; tr-fissures absent.

##### Ecology.

Freshwater, acidophilus, epiphytic species, inhabits moss vegetation, green filamentous algae. In Spain the species was collected in habitats with pH 4.3–7.9, conductivity 4.17–720 μS/cm, the altitude 76–1356 m asl, SPI 12.3–20. Optimum conditions with pH 5.3–6.8, conductivity 28.7–51 μS/cm, the altitude 472–624 m asl, SPI 19.3–19.7 (Ortiz-Lerín and Cambra 2007).

##### Distribution.

EUROPE: Britain, France, Germany, Netherlands (M. Gury in Guiry and Guiry 2019); Macedonia (Pavlov and Levkov 2013), Poland, Romania, Slovakia, Spain (M. Gury in Guiry and Guiry 2019); Ukraine (present paper). AFRICA: Ghana. ASIA: Russia. AUSTRALIA: Australia, New Zealand. N. AMERICA: Canada, USA. S. AMERICA: Argentina, Brazil, Colombia (M. Gury in Guiry and Guiry 2019). **In Ukraine.** The Cheremsky Nature Reserve, tract Obkopane, Lake Redychi, epiphyton on *Fontinalis* sp.

##### Comments.

Illustration of E.impressavar.angusta in Van Heurck (1881: pl. 35/fig. 1) is not conspecific to *E.implicata* sensu Nörpel-Schempp et al. (in Alles et al. 1991) as it has depression on dorsal margin and the poles turned to dorsal valve side. In many literature sources the illustrations of this species are not uniform in valve outline and often do not correspond to the species lectotype.

#### Eunotia incisa

Taxon classificationPlantaeEunotialesEunotiaceae

W. Smith ex Gregory, 1854: p. 96, pl. 4/fig. 4. *

913ced8f-3b2f-5967-aca7-fadf1932faac

Figs 21–23

##### Lectotype.

*Eunotiaincisa* W. Smith ex Gregory, 1854: pl. 4/fig. 4. (= Fig. 22 here), designated here.

##### Illustrations.

Krammer and Lange-Bertalot 1991: p. 221, pl. 161/figs 8–19, pl. 162/figs 1–2 (SEM), pl. 163/figs 1–7; Ohtsuka 2002: fig. 62; Ortiz-Lerín and Cambra 2007: p. 424, figs 5/O-R, 6/C, K; Taylor et al. 2007: pl. 20/5 exemplars; Furey 2011: 7 exemplars and 2 (SEM); Bąk et al. 2012: p. 134, pl. 16/8 exemplars; Ector et al. 2015: p. 254–256, 25 exemplars; Costa 2015: p. 55, pl. 22/figs 1–21, pl. 23/figs 1–5 (SEM); Bahls et al. 2018: pl. 23/figs 19–28, pl. 40/figs 2,3; pl. 87/figs 11, 12; pl. 112/figs 17–19.

##### Diagnosis.

Morphometric data: length 17–27 µm, width 3.5–4.0 µm, striae density 19–22 in 10 µm. Costa 2015: length 18–43 µm, width 3.0–4.5 µm, striae density 18–21 in 10 µm.

Frustule bi-symmetric, bipolar, biraphid with mirror-symmetric, mantle-offset, brevisslit type of raphe, in girdle view rectangular. Valves dorsiventral with convex dorsal, straight ventral margins and gradually contracted acutely rounded poles turned to ventral valve side. Dorsal mantle arcuate with uninterrupted striae; ventral mantle abruptly perpendicular to the valve surface, hyaline, its height is about 0,5 of valve width (see Costa 2015: pl. 23/figs 1, 3, 5). Striae basal, uniserial, distant, gradually compacted from valve center to the poles (see Costa 2015: pl. 23/fig. 1). Areolae small with round outer foramina. Raphe system consists of two short filiform arcuate slits on hyaline area of ventral valve mantle, on external valve surface distal ends of the slits finish by distant from the poles round funnels on valve/mantle junction; central raphe pores are funnel-like; tr-fissures absent. One apical rimoportula has round external opening (see Costa 2015: pl. 23/figs 3, 5).

##### Ecology.

Freshwater benthic species occurs in upland streams in acidic, xeno-oligosaprobic waters with poor electrolytes content (Ortiz-Lerín and Cambra 2007, Taylor et al. 2007). In rivers and streams of Northern Spain has been recorded highest abundance between 7–10% in conditions with pH 5.3–6, conductivity 38–51 μS/cm, altitude 472–484 m asl, SPI 19.3–19.7 (Ortiz-Lerín and Cambra 2007). The species was found both in oligo- and eutrophic waters: total phosphorus < 71.4 mg/cm^3^, conductivity 13–142 μS/cm and pH 5.3–9.3. High abundances of *E.incisa* reported from eutrophic conditions are in disagreement with other literature data (Costa 2015).

##### Distribution.

EUROPE: Baltic Sea, Belgium, Britain, Czech Republic, Finland, France, Germany, Ireland, Italy, Macedonia, Netherlands, Poland, Romania, Russia, Spain (M. Gury in Guiry and Guiry 2019); Ukraine (present paper). N. AMERICA: USA, Canada (M. Gury in Guiry and Guiry 2019). S. AMERICA: Brazil, Colombia. AFRICA: South Africa (Taylor et al. 2007); Ghana, Sudan (M. Gury in Guiry and Guiry 2019). ASIA: India, Israel; Bering Island, Korea, Russia, Singapore (M. Gury in Guiry and Guiry 2019); Japan (Ohtsuka 2002). AUSTRALIA: New Zealand, Australia (M. Gury in Guiry and Guiry 2019). **In Ukraine.** The Cheremsky Nature Reserve, tract Obkopane, Lake Redychi, epiphyton on *Fontinalis* sp.

##### Comments.

Distal ends of the raphe slits are clearly visible on the valve/mantle ridge in LM photos, which is a valuable character in species identification.

#### Eunotia jarensis

Taxon classificationPlantaeEunotialesEunotiaceae

Lange-Bert. et al., 2003: p. 41, pl. 124/figs 7–11. *^

913ced8f-3b2f-5967-aca7-fadf1932faac

Figs 12 , 12a

##### Illustrations.

Pavlov and Levkov 2013: p. 13, pl. 13/figs 1–5.

##### Diagnosis.

Morphometric data: length 63 µm, width 9 µm, striae density c12, p18 in 10 µm. Lange-Bertalot et al. 2003: length 36–76 µm, width 8–10 µm, striae density 10–12 in 10 µm.

Frustule bi-symmetric, bipolar, biraphid with mirror-symmetric, mantle-offset, brevisslit type of raphe. Valves dorsiventral, uniform in width, with two very weak undulations on dorsal margin, weakly concave ventral margin and protracted broadly rounded poles slightly deflected to dorsal side. Striae basal, uniserial, distant, evenly spaced. Areolae small with round foramina (Fig. 12a). Raphe system consists of two short filiform slits on ventral valve mantle; helictoglossae average in size; one rimoportula present at the middle of pole (Fig. 12a).

##### Ecology.

Freshwater species, epiphytic on the moss, at an altitude of about 2300 m a.s. (Pavlov and Levkov 2013).

##### Distribution.

EUROPE: **Type locality**: ITALY, Pauli Murdegu, Insula Sardinia (Lange-Bertalot et al. 2003); North Macedonia, Shara Mountain, stream above glacial Lake Crno (Pavlov and Levkov 2013); Ukraine (present paper). **In Ukraine.** The Cheremsky Nature Reserve, tract Obkopane, ditch, epiphyton on *Sphagnum* sp.

##### Type information.

25.05.1994, leg. A. Bardi, (Praep. No. Eu-I-159 in Coll. Lange-Bertalot, Botan. Institut Universität Frankfurt a.M.).

##### Comments.

Raphe system has not been studied from outer valve surface in SEM but this species certainly does not have tr-fissures which are always situated on a hyaline field (Fig. 12a) and distal ends of the raphe slits finish on ventral valve margin at the poles, similar to *E.dorofeyukiae* (Fig. 27a).

Most specimens in the population from North Macedonia differ through having much narrower poles (Pavlov and Levkov 2013: pl. 13/figs 3–6) than in the type population.

Very rare species occurs only in Europe in three localities in low abundance.

#### Eunotia julma

Taxon classificationPlantaeEunotialesEunotiaceae

Lange-Bert. in Lange-Bertalot et al. 2011: pl. 7/figs 1–7, 8–10. *^

913ced8f-3b2f-5967-aca7-fadf1932faac

Figs 7 , 8 , 8a (SEM)

##### Holotype.

Lange-Bertalot et al. 2011: pl. 7/fig. 1, designated by Lange-Bertalot in Lange-Bertalot et al. 2011.

##### Illustrations.

Potapova et al. 2014: fig. 1; Kulikovskiy et al. 2016: p. 122, pl. 27/figs 14–17; Bouchard et al. 2018: pl. 1/fig. 1.

##### Diagnosis.

Morphometric data: length 115–175 µm, width 6 µm, striae density c12, p16 in 10 µm. Lange-Bertalot et al. 2011: length 70–150 µm, 4,5–5 µm, striae density c14, p16 in10 µm.

Frustule bi-symmetric, bipolar, biraphid with mirror-symmetric, mantle-offset, brevisslit type of raphe. Valves dorsiventral, uniform in width, arcuate, with rounded poles. Striae basal, uniserial, distant, evenly spaced (Figs 7, 8, 8a). Areolae small with round outer foramina. Raphe system consists of two short filiform slits on ventral valve mantle; tr-fissures curved on the valve surface, pass along four striae on the middle of valve and end up by round lacunae (Fig. 8a).

##### Ecology.

Freshwater epiphytic species.

##### Distribution.

EUROPE: **Type locality**: Finland, Lake Julma Olkky near Kuusamo (Lange-Bertalot et al. 2011); Netherlands (M. Gury in Guiry and Guiry 2019); Ukraine (present paper). ?ASIA: Russia, Eastern Siberia (Potapova et al. 2014); Russia (Kulikovskiy et al. 2016). N. AMERICA: Canada (Bouchard et al. 2018). **In Ukraine.** The Cheremsky Nature Reserve, tract Obkopane, ditch, epiphyton on *Sphagnum* sp.

##### Comments.

In primary description it is indicated that “ … all specimens are consistently curved” (Lange-Bertalot et al. 2011). Our exemplars correspond to the species Holotype in valve outline and morphometry except our specimens are longer and wider. The illustrations of *E.julma* in Potapova et al. (2014: fig. 1), Kulikovskiy et al. (2016: pl. 27/figs 14–17), Bouchard et al. (2018: pl. 1/fig. 1) differ from the Holotype by almost straight valves.

#### Eunotia neocompacta

Taxon classificationPlantaeEunotialesEunotiaceae

S. Mayama in Mayama and Kawashima 1998: p. 69.

913ced8f-3b2f-5967-aca7-fadf1932faac

Figs 30 , 30a (SEM)

Eunotia exigua var. compacta Hustedt, 1930: p. 176, fig. 225 [Basionym]
Eunotia compacta (Hustedt) S. Mayama, 1997
Eunotia neocompacta var. vixcompacta Lange-Bert. in Lange-Bertalot et al. 2011

##### Illustrations.

Grunow in Van Heurck 1881: pl. 34/fig. 8 – second exemplar; Krammer, Lange-Bertalot 1991: pl. 134/figs 32, 35–38 (both citations as *Eunotianymanniana* Grunow). Mayama 1997: p. 35, figs 22–25, 26–31 (SEM) (as *E.compacta*). Lange-Bertalot et al. 2011: p. 173–174, pl. 123/figs 1, 2–16, 25–28, 32–34 (SEM); Bąk et al. 2012: pl. 19/1, 2 exemplars from left to right; Genkal and Komulaynen 2015: fig. 2л; Rivera-Rondón and Catalan 2017: pl. 33/figs 15–19 (all citations as E.neocompactavar.vixcompacta). Kulikovskiy et al. 2016: p. 126, pl. 25/figs 7–11; Mimura and Ohtsuka 2016: fig. 34; Bahls et al. 2018: pl. 83/figs 8, 9.

##### Diagnosis.

Morphometric data: length 27 µm, width 3.5 µm, striae density 20 in 10 µm.

Mayama 1997: length 18–57 µm, width 3.5–5 µm, striae density 20–22 in 10 µm.

Frustule bi-symmetric, bipolar, biraphid with mirror-symmetric, mantle-offset, brevisslit type of raphe. Valves dorsiventral, weakly arcuate, uniform in width, with truncated poles strongly deflected to dorsal side. Striae basal, uniserial, distant, evenly spaced (Fig. 30a). Areolae small with round outer foramina. Raphe system consists of two short filiform slits which are straight on ventral valve mantle and widely rounded at valve poles, distal ends of the slits finish on external valve surface on the middle of the poles by small round pores connected with helictoglossae of average size (Fig. 30a; see Mayama 1997: figs 28, 29); central raphe pores on outer valve surface are funnel-like (see Mayama 1997: fig. 31); tr-fissures absent (see Mayama 1997: fig. 31).

##### Ecology.

Freshwater epiphytic species.

##### Distribution.

EUROPE: Georgia, Ireland, Netherlands, Poland, Romania, Ukraine (M. Gury in Guiry and Guiry 2019); France, Pyrenees, Lake Monges (Rivera-Rondón and Catalan 2017); Russia (Genkal and Komulaynen 2015: as E.neocompactavar.vixcompacta). N. AMERICA: USA, Alaska, Atlantic Islands (M. Gury in Guiry and Guiry 2019); Canada (Bahls et al. 2018). ASIA: Japan (Mimura and Ohtsuka 2016); Russia (Kulikovskiy et al. 2016); Russia, Bering Island (M. Gury in Guiry and Guiry 2019). **In Ukraine.** First record in the Cheremsky Nature Reserve, tract Obkopane, Lake Redychi, epiphyton on *Sphagnum* sp.

##### Comments.

In some publications the illustrations of this species are not uniform in valve outline, therefore only those microphotos which correspond to the species concept in Mayama (1997) are cited in the present paper. For instance, the specimen in Bouchard et al. (2018: pl. 1/fig. 6) has arcuate valve and longer poles therefore does not match to *E.neocompacta* sensu stricto.

Based on its morphology, Eunotianeocompactavar.vixcompacta (Lange-Bertalot et al. 2011) is conspecific with *E.neocompacta*, which has also been confirmed by other authors (Kulikovskiy et al. 2016, Mimura and Ohtsuka 2016, Bahls et al. 2018).

#### Eunotia praerupta

Taxon classificationPlantaeEunotialesEunotiaceae

Ehrenb., 1843: p. 414.

913ced8f-3b2f-5967-aca7-fadf1932faac

Fig. 31 (SEM)

Eunotia praerupta f. curta (Grunow) Mayer, 1917
Eunotia bidens var. praerupta (Ehrenb.) Aysel, 2005

##### Illustrations.

Krammer and Lange-Bertalot 1991: pl. 148/figs 1–3; Pavlov and Levkov 2013: p. 35, pl. 18/fig. 7 (SEM), pl. 19/figs 1–10.

##### Diagnosis.

Morphometric data: length 73 µm, width 20 µm; striae density c5, p8 in 10 µm. Pavlov and Levkov 2013: length 37–73 µm, width 13–17 µm; striae density c5–9, p8–12 in 10 µm.

Frustule bi-symmetric, bipolar, biraphid with mirror-symmetric, mantle-offset, brevisslit type of raphe. Valves dorsiventral, with strongly convex dorsal and weakly concave ventral margins, gradually narrowed to rostrate poles that are about perpendicular to the valve margins. Striae basal, uniserial, distant, irregularly spaced. Areolae small with round outer foramina. Raphe system consists of two short filiform slits on ventral valve mantle, distal ends of the slits finish on external valve surface on about 0.3 of pole width by small round pore-like outer connected with helictoglossae (see Pavlov and Levkov 2013: pl. 18/fig. 7); tr-fissures absent.

##### Ecology.

Freshwater epiphytic species.

##### Distribution.

Species was recorded in most European countries and on all continents except Antarctica (M. Gury in Guiry and Guiry 2019). **In Ukraine.** First record in the Cheremsky Nature Reserve, tract Obkopane, ditch, epiphyton on *Sphagnum* sp.

##### Comments.

No illustrations were published by the author of this species, which has led to a very wide species concept and uncertain taxonomy. In this paper the concept of *Eunotiapraerupta* sensu stricto proposed in Krammer and Lange-Bertalot (1991: pl. 148/figs 1–3) has been followed.

#### Eunotia pseudoflexuosa

Taxon classificationPlantaeEunotialesEunotiaceae

Hustedt, 1949: p. 71, pl. 2/figs 16–18. *^

913ced8f-3b2f-5967-aca7-fadf1932faac

Figs 13 , 13a

##### Illustrations.

Simonsen 1987: p. 340, pl. 522/figs 1–6; Kulikovskiy et al. 2010b: pl. 26/figs 1, 2, 4, 5 (LM), 3, 6, 7 (SEM); Kulikovskiy et al. 2016: pl. 26/figs 5–8; Bahls et al. 2018: pl. 83/fig. 1, pl. 112/fig. 6.

##### Diagnosis.

Morphometric data: length 112 µm, width 4 µm, striae density 11 in 10 µm.

Kulikovskiy et al. 2016: length 80–180 µm, width 4–6 µm, striae density c11–14, p15–20 in 10 µm.

Frustule bi-symmetric, bipolar, biraphid with mirror-symmetric, mantle-offset, brevisslit type of raphe. Valves dorsiventral, uniform in width, with subcapitate poles deflected to dorsal side. Striae basal, uniserial, distant, evenly spaced (Fig. 13a). Areolae small with round outer foramina. Raphe system consists of two short filiform slits on ventral valve mantle; tr-fissures long, occupy 7–8 striae in the middle of valve width; helictoglossa average in size (Fig. 13a).

##### Ecology.

Freshwater epiphytic species.

##### Distribution.

AFRICA: **Type location**: [Democratic Republic of the Congo, Virunga National Park], vulkan region, Lake Karisimbi. EUROPE: Russia (Kulikovskiy et al. 2016); Ukraine (present paper). ASIA: Mongolia (Kulikovskiy et al. 2010b); Russia (Kulikovskiy et al. 2016). N. AMERICA: Canada (Bahls et al. 2018); USA, Alaska (M. Gury in Guiry and Guiry 2019). **In Ukraine.** The Cheremsky Nature Reserve, tract Obkopane, ditch, epiphyton on *Sphagnum* sp.

##### Type information.

„Albert-National park in Belgisch-Kongo.”

#### Eunotia ruzickae

Taxon classificationPlantaeEunotialesEunotiaceae

Bílý & Marvan, 1962: p. 293, figs 1–5. *^

913ced8f-3b2f-5967-aca7-fadf1932faac

Fig. 14

##### Illustrations.

Pavlov and Levkov 2013: pl. 58/fig. 7.

##### Diagnosis.

Morphometric data: length 44 µm, width cp6, m7 µm; striae density c16, p22 in 10 µm. Bílý and Marvan 1962: length 40–90 µm, width 4.5–6 µm, striae density 13–14 in 10 µm.

Frustule bi-symmetric, bipolar, biraphid with mirror-symmetric, mantle-offset, brevisslit type of raphe. Valves dorsiventral, uniform in width, with slightly undulate dorsal margin and weak depression in its central part, usually straight ventral side, sometimes with weak central convexity (see Bílý and Marvan 1962: fig. 1), and broad rounded poles deflected to dorsal side. Striae basal, uniserial, distant, denser at the poles, irregularly spaced. Raphe system consists of two short filiform slits on ventral valve mantle, distal ends of the slits terminate at the poles about 0.3 of valve width from ventral margin.

##### Ecology.

Freshwater epiphytic species.

##### Distribution.

EUROPE: **Type location**: Czech Republic (Bílý and Marvan 1962); North Macedonia, (Pavlov and Levkov 2013); Germany, Scandinavia (M. Guiry in Guiry and Guiry 2019); Ukraine (present paper). **In Ukraine.** Zhytomyr region, Chervonoarmeisky district, swamp, dark films among mosses.

##### Type information.

“In bentho piscinae Řežabinec prope vicum Ražice in Bohemia meridionali atque in nonnullis locis Moraviae merdionalis”, (Typus in herbario Inst. bot. Univ. Brunensis, Brno).

##### Comments.

The found exemplar has denser striae than in type population. This species has typical raphe system without terminal raphe fissures, however SEM illustrations of the raphe to confirm this were not found.

#### Eunotia tetraodon

Taxon classificationPlantaeEunotialesEunotiaceae

Ehrenberg, 1838: p. 192, pl. 21/fig. 25.

913ced8f-3b2f-5967-aca7-fadf1932faac

Figs 34 , 35

Himantidium tetraodon (Ehrenb.) Bréb. ex Kützing, 1849
Eunotia robusta var. tetraodon (Ehrenb.) Ralfs, 1861
Eunotia diadema var. tetraodon (Ehrenb.) A. Cleve, 1953
Eunotia serra var. tetraodon (Ehrenb.) Nörpel in Krammer and Lange-Bertalot 1991

##### Illustrations.

Topachevsky and Oksiyuk 1960: p. 323, pl. 119/fig. 1 (as *Eunotiarobusta* Ralfs); Bahls 2012: 6 exemplars; Bąk et al. 2012: pl. 15/4 exemplars; Ector et al. 2012: p. 270–271, 1 exemplar; Pavlov and Levkov 2013: pl. 16/figs 1–9, 10, 11 (SEM); Kulikovskiy et al. 2016: p. 133, pl. 29/figs 6–10; Malakhov et al. 2017: p. 67, fig. 10 (SEM); Bahls et al. 2018: pl. 21/fig. 2.

##### Diagnosis.

Morphometric data: length 40 µm, width c8–10, m10–13, p4–8 µm; striae density c11–15, p16 in 10 µm. Pavlov and Levkov 2013: length 25–62 µm, width 9.5–16 µm, striae density 6–10, m12–16 in 10 µm.

Frustule bi-symmetric, bipolar, biraphid with mirror-symmetric, mantle-offset, brevisslit type of raphe. Valves dorsiventral, with strongly convex, four-times strongly undulate dorsal and weakly concave ventral margins, gradually narrowed to the protracted poles that continue the dorsal arc of valve margin. Striae basal, uniserial, distant, irregularly spaced, on dorsal side shortened intermediate striae present. Areolae small with round outer foramina. Raphe system consists of two short filiform slits on ventral valve mantle that follow pole margin and finish on about 0.5 of pole width by small round pore (see Pavlov and Levkov 2013: pl. 26/fig. 11) connected with helictoglossae; tr-fissures absent.

##### Ecology.

Freshwater epiphytic species.

##### Distribution.

Species was recorded in most European countries and on all continents except Antarctica (M. Gury in Guiry and Guiry 2019). **In Ukraine.** Volyn region, Manevychi district, Lake Bile; Rivnenska region, Bog Gala (Topachevsky and Oksiyuk 1960 – as *Eunotiarobusta* Ralfs); Volyn region, the Cheremsky Nature Reserve, Lake Redychi (Malakhov et al. 2017); Lake Redychi, epiphyton on *Fontinalis* sp.

**Comments.** In Ukraine this species is quite rare. Only five reliable records exist that were accompanied by illustrations, including this paper, and all are from Ukrainian Polissya and Carpaty. Initially this species was reported by Topachevsky and Oksiyuk (1960) as *E.robusta* however their illustration corresponds to *E.tetraodon*. The next reports came almost 60 years later (Malakhov et al. 2017, present paper).

#### Eunotia

Taxon classificationPlantaeEunotialesEunotiaceae

sp. 1 (cf. Eunotia formica Ehrenberg, 1843: p. 414)

913ced8f-3b2f-5967-aca7-fadf1932faac

Fig. 9

##### Morphometric data.

Length 117 µm, width c8, m6, p10 µm; striae density c9, p11 in 10 µm.

##### Distribution in Ukraine.

The Cheremsky Nature Reserve, tract Obkopane, Lake Redychi, epiphyton on *Sphagnum* sp.

##### Comments.

This specimen is most similar to *E.formica* which is widely distributed and has been found on all continents except Antarctica (Gury M in Guiry and Guiry 2019). The original illustrations of *E.formica* by Ehrenberg were not introduced in primary description and the species concept in literature is different from our exemplar, which has narrower valves and rhombic poles (see Krammer and Lange-Bertalot 1991: p. 209, pl. 152/8–12a; Lange-Bertalot and Metzeltin 1996: p. 144, pl. 13/1, 2; Ortiz-Lerín and Cambra 2007: p. 424, fig.3/T; Taylor et al. 2007: pl. 22/figs 1, 3).

#### Eunotia

Taxon classificationPlantaeEunotialesEunotiaceae

sp. 2 (cf. Eunotia mongolica Kulikovskiy et al. 2010b: p. 124, pl. 40/figs 1–5)

913ced8f-3b2f-5967-aca7-fadf1932faac

Figs 17 , 17a (SEM)

##### Illustrations.

Kulikovskiy et al. 2016: p. 124, pl. 28/figs 26–30.

##### Morphometric data.

Length 73 µm, width 3.5 µm, striae density c16, p20 in 10 µm. Kulikovskiy et al. 2016: length 35–55, width 2.3–2.7, striae density c19, p23.

##### Distribution in Ukraine.

The Cheremsky Nature Reserve, tract Obkopane, Lake Redychi, epiphyton on *Sphagnum* sp.

##### Comments.

This specimen is similar to *E.mongolica* in valve outline, however it differs in metric parameters and fine morphology, having shorter tr-fissures (Fig. 17a).

#### Eunotia

Taxon classificationPlantaeEunotialesEunotiaceae

sp. 3 (cf. Eunotia paludosa Grunow, 1862: p. 336, pl. 3/fig. 10a–d)

913ced8f-3b2f-5967-aca7-fadf1932faac

Figs 18–20

##### Morphometric data.

Length 19–24 µm, width 3.5–4 µm; striae density c14–16, p22 in 10 µm.

##### Distribution in Ukraine.

The Cheremsky Nature Reserve, tract Obkopane, ditch, epiphyton on *Sphagnum* sp.

##### Comments.

These specimens correspond to the current literature concept of *E.paludosa* in valve outline of small specimens (see Furey 2012: figs 4–6 from left; Pavlov and Levkov 2013: pl. 42/figs 23–38; Costa 2015: pl. 17/figs 1–10; Kulikovskiy et al. 2016: pl. 19/figs 8, 9). However, the illustrations by Grunow (1862: p. 336, pl. 3/figs 10a–c) show a much more arcuate valve outline that differs significantly from the literature concept.

The other species whose small specimens have similar valve outline to the discovered specimens was reported as *Eunothiafennica* (Hustedt) Lange-Bert. in Werum and Lange-Bertalot (2004) by Noga (2019: only figs 2e–g, 3c–e). However, the detail study of *E.fennica* (Hamilton and Siver 2010: figs 1–11, 16–29) shows a clear difference from the specimens on Figs 17–19 and the ones, cited from Noga (2019): in valve outline, presence of spines, morphonetric data. Hamilton and Siver (2010) also have underlined that *E.fennica* can be confused with *E.paludosa*, more over, the authors found both species in the same sample.

In Topachevsky and Oksiyuk (1960)*E.paludosa* was considered a synonym of *Eunotiaexigua* (Bréb. ex Kütz.) Rabenh. but both species are valid taxa at present.

#### Eunotia

Taxon classificationPlantaeEunotialesEunotiaceae

sp. 4 (cf. Eunotia intermedia (Krasske ex Hustedt) Nörpel et al. 1993: p. 32)

913ced8f-3b2f-5967-aca7-fadf1932faac

Fig. 24

##### Morphometric data.

Length 23 µm, width 4 µm, striae density 11 in 10 µm.

##### Distribution in Ukraine.

Volyn region, Vladimir-Volyn district, near village Fedorovka, Western Bug River, floodplain basin, benthos.

##### Comments.

This exemplar has more curved valves and non-narrowed poles and differs in valve outline from *E.intermedia* (see Krammer and Lange-Bertalot 1991: p. 215, pl. 143/figs 11, 13; Jüttner et al. 2019: 7 exemplars).

#### Eunotia

Taxon classificationPlantaeEunotialesEunotiaceae

sp. 5 (cf. Eunotia meridiana Metzeltin & Lange-Bertalot, 1998: p. 67, pl. 59/figs 7–10).

913ced8f-3b2f-5967-aca7-fadf1932faac

Figs 25–27

##### Morphometric data.

Length 17–18 µm, width 3–3.5 µm, striae density 21 in 10 µm.

##### Distribution in Ukraine.

The Cheremsky Nature Reserve, tract Obkopane, Lake Redychi, epiphyton on *Sphagnum* sp

##### Comments.

These exemplars differ from *E.meridiana* in valve outline, narrower valves and higher striae density.

#### Eunotia

Taxon classificationPlantaeEunotialesEunotiaceae

sp. 6.

913ced8f-3b2f-5967-aca7-fadf1932faac

Fig. 32

##### Morphometric data.

Length 70 µm, width 12 µm, striae density c7, p11 in 10 µm.

##### Distribution in Ukraine.

The Cheremsky Nature Reserve, tract Obkopane, ditch, epiphyton on *Sphagnum* sp.

#### Eunotia

Taxon classificationPlantaeEunotialesEunotiaceae

sp. 7.

913ced8f-3b2f-5967-aca7-fadf1932faac

Fig. 33

##### Morphometric data.

Length 40 µm, width 12 µm, striae density 10 in 10 µm.

##### Distribution in Ukraine.

The Cheremsky Nature Reserve, tract Obkopane, ditch, epiphyton on *Sphagnum* sp.

##### Comments.

Both *Eunotia* sp. 6 and *Eunotia* sp. 7 have similar morphology, valve width and striae density. Moreover, both exemplars were found in the same sample, which may suggest that they belong to the same species, but insufficient data does not provide a conclusion at present.

## Discussion and conclusions

The genus *Eunotia* is one of the largest within the Order Bacillariophyta and totals 589 valid taxa. This can be considered as an evolutionary success of the genus, relevant to the frustule morphology that is well suited to the ecological conditions where the *Eunotia* species inhabit.

The presence of raphe system is certainly a progressive feature which has appeared in the diatom frustule evolution since the number of species bearing it exceeds significantly the ones without raphe.

The complicated morphology of *Eunotia* species has led to the numerous synonyms – more than 60% of taxonomic names (Guiry M in Guiry and Guiry 2019). Therefore it is a necessary task to find reliable morphological characters which can be useful in species identification and description.

The genus *Eunotia* possesses of mirror-symmetric, mantle-offset, brevisslit raphe system the combination of the characters in which is unique among diatom genera. At the same time different *Eunotia* species have peculiar details in the raphe system which belong to species rank of taxonomy: presence / absence of tr-fissures and their shape, shape of raphe slits and their position on the valve etc. (Table 1).

**Table 1.** Key morphological features in the genus *Eunotia* to recognize studied species. Abbreviations: **dd-pore** difficultly distinguishing pores, **DS** dorsal side, **VM** ventral mantle, **VM+V** ventral mantle + valve surface, **VP** valve pole, – absent, **US** unstudied

**Species**	**Valve outline**			**Kind of striae**	**Striae arrangement**	**Raphe system on outer valve surface**						
	**Dorsal margin**	**Ventral margin**	**Shape of the poles**			**Shape of slits**	**Dispo- sition of slits**	**Shape of distal ends of the slits**	**Disposition of distal ends of the slits**	**Terminal fissures shape**	**Disposition of terminal fissures**	**Shape of central pores**
*** E. dorofeyukiae ***	undulate	slightly concave	subcapitate, broad rounded	dis-tant	denser at the poles	US	VM+V	small round funnels	ventral VP corners	–	–	US
*** E. implicata ***	weakly convex	concave	protracted, rounded	dis- tant	irregularly spaced, denser at the poles	US	VM+V	turned to the valve centre under right corner, finish by small round pores	ventral VP corners	–	–	US
*** E. incisa ***	weakly convex	Straight	gradually contracted acutely rounded	dis- tant	gradually compacted to the poles	arcuate	VM	round funnels	ventral margin	–	–	funnel-like
*** E. jarensis ***	uniform width, weakly convex	weakly concave	protracted, broadly rounded	dis- tant	evenly spaced	US	VM+V	US	ventral VP corners	US	US	US
*** E. neocompacta ***	uniform width, weakly convex	weakly concave	truncated, strongly deflected to DS	dis- tant	evenly spaced	straight on VM, rounded at VP	VM+V	small round pores	middle of VP	–	–	funnel-like
*** E. praerupta ***	strongly convex	weakly concave	rostrate	dis- tant	irregularly spaced		VM+V	small round pores	ventral VP corners	–	–	US
*** E. ruzickae ***	slightly undulate	straight	broad round, deflected to DS	dis- tant	irregularly spaced, compacted to the poles	US	VM+V	US	ventral VP corners	US	US	US
*** E. tetraodon ***	strongly convex, four-times strongly undulate	weakly concave	protracted,	dis- tant	irregularly spaced,	US	VM+V	small round pores	middle of VP	–	–	US
*** E. formicina ***	weakly convex	weakly concave, gentle gibbosity	subcapitate, broad rounded	dis- tant	irregularly spaced	US	VM+V	dd-pores	ventral VP corners	long, round	follow poles outline, finish on DM	round
*** E. genuflexa ***	weakly convex	weakly concave	narrowed rounded	dis- tant	evenly spaced	US	VM+V	dd-pores	ventral VP corners	long, round, with lacunae	mid-valve	US
*** E. julma ***	uniform width, convex	concave	round	dis- tant	evenly spaced	US	VM+V	dd-pores	–	long, round, with lacunae	mid-valve	US
*** E. pseudoflexuosa ***	uniform width, weakly convex	weakly concave	subcapitate, deflected to DS	dis- tant	evenly spaced	US	VM+V	US	US	long	mid-valve	US

The morphological analysis carried out in this study revealed that 8 of 12 studied species of *Eunotia* do not have terminal raphe fissures (Table 1). Instead, the distal ends of the raphe slits terminate on the outer valve surface by pore-like (Figs 3c, 28a, 30) or funnel holes (Fig. 3d, see Mayama 1997: fig. 31) which are connected with helictoglossae. However, in the literature those distal ends of the raphe slits were erroneously described as tr-fissures (e.g. Pavlov and Levkov 2013: p. 20, pl. 18/fig. 7; p. 43, pl. 26/fig. 11). *Eunotiakrammeri* Kulikovskiy et al. also has a raphe system without tr-fissures that is clearly visible on SEM illustrations both from external and internal valve surfaces (Kulikovskiy et al. 2010a: p. 102, pl. 7/figs 18, 19). Nevertheless, the authors report them in their description of this species.

In morphology of *Eunotia* the characters suitable for the species identification are quite restricted. Besides, the frustule ultrastructure is poorly studied even for the species which were investigated with SEM, in particularly, raphe system. For many species there is still no data on the slits form, central pores etc. In the meantime, among key diagnostic characters for the *Eunotia* species identification more than half include the ones which refer to the peculiarity of raphe system (Table 1). For instance, central raphe pores usually have a different shape on the inner and outer valve surfaces, however their shape on inner surface is uniform within the genera and some taxa of higher rank of taxonomy while the shape of central pores on the outer valve surface has the species rank of taxonomy (Bukhtiyarova and Pomazkina 2013).

Thus, careful application of suggested terms in future is necessary when describing new *Eunotia* taxa and will be helpful in correct identification of the known species.

Species of *Eunotia* prefer acidic, dystrophic or oligotrophic freshwater habitats, mostly of low conductivity and usually inhabit in epiphytic or epilithic hydrotopes (Alles et al. 1991, Metzeltin and Lange-Bertalot 1998, Siver et al. 2006, Cantonati and Lange-Bertalot 2011, Pavlov and Levkov 2013, Bahls et al. 2018). In Ukraine 32 species and eight varieties of *Eunotia* were known until this study and now 9 more species are reported for the first time. Thus, the total number of *Eunotia* species in Ukraine is 41, which is only 7% of the species in this genus worldwide. This is indirect evidence of insufficient investigation of the wetlands in Ukraine where *Eunotia* has high species richness. The findings in the present study include five species widely distributed in the world flora on most continents and seven rare species that are known from several locations, among the latter are *E.genuflexa, E.jarensis* and *E.ruzickae*, which are probably European endemics. At present, in the Cheremsky Nature Reserve, 20 species have been recorded, which is the largest number of *Eunotia* species in any region of Ukraine. In total 19 *Eunotia* species were recorded in this study together with the ones which were not identified to the species level. Given the large number of poorly studied oligotrophic lakes and bogs in the country, especially in Ukrainian Polissya, it is possible to predict the future discovery of many more *Eunotia* species from Ukraine.

## Supplementary Material

XML Treatment for Eunotia dorofeyukiae

XML Treatment for Eunotia formicina

XML Treatment for Eunotia genuflexa

XML Treatment for Eunotia implicata

XML Treatment for Eunotia incisa

XML Treatment for Eunotia jarensis

XML Treatment for Eunotia julma

XML Treatment for Eunotia neocompacta

XML Treatment for Eunotia praerupta

XML Treatment for Eunotia pseudoflexuosa

XML Treatment for Eunotia ruzickae

XML Treatment for Eunotia tetraodon

XML Treatment for Eunotia

XML Treatment for Eunotia

XML Treatment for Eunotia

XML Treatment for Eunotia

XML Treatment for Eunotia

XML Treatment for Eunotia

XML Treatment for Eunotia
